# L1 chimeric transcripts are expressed in healthy brain and their deregulation in glioma follows that of their host locus

**DOI:** 10.1093/hmg/ddac056

**Published:** 2022-03-17

**Authors:** Marie-Elisa Pinson, Franck Court, Aymeric Masson, Yoan Renaud, Allison Fantini, Ophélie Bacoeur-Ouzillou, Marie Barriere, Bruno Pereira, Pierre-Olivier Guichet, Emmanuel Chautard, Lucie Karayan-Tapon, Pierre Verrelle, Philippe Arnaud, Catherine Vaurs-Barrière

**Affiliations:** Université Clermont Auvergne, CNRS, Inserm, iGReD, Clermont-Ferrand F-63000, France; Université Clermont Auvergne, CNRS, Inserm, iGReD, Clermont-Ferrand F-63000, France; Université Clermont Auvergne, CNRS, Inserm, iGReD, Clermont-Ferrand F-63000, France; Université Clermont Auvergne, CNRS, Inserm, iGReD, Clermont-Ferrand F-63000, France; Université Clermont Auvergne, CNRS, Inserm, iGReD, Clermont-Ferrand F-63000, France; Université Clermont Auvergne, CNRS, Inserm, iGReD, Clermont-Ferrand F-63000, France; Université Clermont Auvergne, CNRS, Inserm, iGReD, Clermont-Ferrand F-63000, France; Biostatistics Department, Délégation à la Recherche Clinique et à l’Innovation, Clermont-Ferrand Hospital, Clermont-Ferrand 63003, France; Cancer Biology Department, CHU de Poitiers, Poitiers 86021, France; Université Clermont Auvergne, INSERM, U1240 IMoST, Clermont-Ferrand 63011, France; Pathology Department, Centre Jean PERRIN, Clermont-Ferrand 63011, France; Cancer Biology Department, CHU de Poitiers, Poitiers 86021, France; INSERM, U1084, Poitiers 86021, France; Université de Poitiers, Poitiers 86000, France; INSERM, U1196 CNRS UMR9187, Curie Institute, Orsay 91405, France; Radiotherapy Department, Curie Institute, Paris 75005, France; Université Clermont Auvergne, Clermont-Ferrand 63000, France; Université Clermont Auvergne, CNRS, Inserm, iGReD, Clermont-Ferrand F-63000, France; Université Clermont Auvergne, CNRS, Inserm, iGReD, Clermont-Ferrand F-63000, France

## Abstract

Besides the consequences of retrotransposition, long interspersed element 1 (L1) retrotransposons can affect the host genome through their antisense promoter. In addition to the sense promoter, the evolutionarily recent L1 retrotransposons, which are present in several thousand copies, also possess an anti-sense promoter that can produce L1 chimeric transcripts (LCT) composed of the L1 5′ UTR followed by the adjacent genomic sequence. The full extent to which LCT expression occurs in a given tissue and whether disruption of the defense mechanisms that normally control L1 retrotransposons affects their expression and function in cancer cells, remain to be established. By using CLIFinder, a dedicated bioinformatics tool, we found that LCT expression was widespread in normal brain and aggressive glioma samples, and that approximately 17% of recent L1 retrotransposons, from the L1PA1 to L1PA7 subfamilies, were involved in their production. Importantly, the transcriptional activities of the L1 antisense promoters and of their host loci were coupled. Accordingly, we detected LCT-producing L1 retrotransposons mainly in transcriptionally active genes and genomic loci. Moreover, changes in the host genomic locus expression level in glioma were associated with a similar change in LCT expression level, regardless of the L1 promoter methylation status. Our findings support a model in which the host genomic locus transcriptional activity is the main driving force of LCT expression. We hypothesize that this model is more applicable when host gene and LCT are transcribed from the same strand.

## Introduction

Long interspersed nuclear element 1 (LINE-1 or L1) is a class of autonomous transposable elements that is mobilized *via* an RNA intermediate by a copy-and-paste mechanism named retrotransposition. L1 retrotransposons represent ~17% of the human genome (1), and 516 000 copies have been annotated in the human reference genome. Seven thousands of them are full length (~6 kb) L1 retrotransposons among which only ~100 can still retrotranspose *in vivo* and belong to the most recent evolutionary L1 subfamily, specific to the human species (L1Hs or L1PA1 elements) (2).

Besides the consequences of retrotransposition (e.g*.* insertional mutagenesis, creation of new alternative splicing sites, promoting sequence transduction from the donor to new insertion sites) (3), L1 retrotransposons can also interfere with the transcriptional activity of the surrounding genomic sequences from their bidirectional promoter. Specifically, in addition to the sense promoter, the 5′ UTR of evolutionarily recent L1s also contain an anti-sense promoter (L1-ASP) that can produce transcripts from the 5′ UTR in antisense orientation to the adjacent genomic region. These transcripts are called L1 chimeric transcripts (LCTs). The L1-ASP was initially described in the L1PA1 subfamily (4), but sequence homology and functional analyses suggest that the L1-ASP appeared first in the L1PA6 subfamily and is active in the more recent subfamilies (L1PA1 to L1PA6) (5,6). L1-ASP activity has been detected also in older L1 families, up to L1PA8, despite the lower sequence homology (6,7). Moreover, it has been demonstrated that the L1 5′ UTR transcribed in the antisense orientation from the L1-ASP contains an open reading frame termed ORF0 (8). Its translation gives rise to a short peptide, or more rarely, to fusion proteins with proximal exons (8). This suggests that at least some LCTs might encode proteins.

In many cancer types, disruption of the defense mechanisms that normally control L1 retrotransposons promotes their expression and mobilization (9,10). Specifically, cancer-associated aberrant DNA hypomethylation of the L1 5′ UTR correlates with L1 transcriptional activation (10,11). Therefore, it has been proposed that L1 retrotransposons can contribute to oncogenesis by somatic neo-transposition (that can be a driver event) (11,12), and also by aberrant LCT transcription, a phenomenon called ‘onco-exaptation’ (13). For instance the *L1-MET* LCT initiates in the second intron of the gene encoding the tyrosine protein kinase MET (*c-MET*) (14). In various cancer types, *L1-MET* LCT expression is inversely correlated with methylation of its L1 5′ UTR, and might have oncogenic functions (15–18). Similarly, it has been suggested that LCT13, a tumor-specific long non-coding LCT, induces repression of the 300 kb distant tumor suppressor gene *tissue factor pathway inhibitor 2* (*TFPI-2*) in various malignancies (19).

These observations stress that recent L1s (L1PA1 to PA8) with a full length 5′ UTR represent an abundant source of alternative promoters that can disrupt the expression of nearby genes in cancer. This highlight the need to identify LCTs in a systematic manner in the different cancer types. Until now, LCTs have been characterized mainly using bioinformatics approaches. For instance, dbEST has been queried to identify spliced ESTs in which an L1 5′ UTR sequence in antisense orientation is spliced with a gene exon (4,7,14,17,18). In 2009, Cruickshanks and Tufarelli (20) developed a dedicated molecular approach (called L1 Chimera Display) that allowed identifying 18 LCTs (intronic or intergenic) in breast cancer samples and cell lines. Altogether, these studies identified 161 recent L1 retrotransposons implicated in the production of LCTs that are expressed in various normal tissues and/or cancer types or cell lines, and among which some might be cancer-specific (19,20). However, these studies had very specific experimental criteria (position of the L1 primer in the LCT and selection only of spliced exon-containing LCT ESTs) and limitations (i.e*.* absence of weakly expressed transcripts in EST libraries). Therefore, to gain insights into the LCT expression profile in a given tissue and into its relevance to cancer development and progression, we and others developed tools to identify transposable element-derived chimeric transcripts from RNA-seq data. These tools include the LIONS suite (21), a tool to identify transposable element-derived oncogene transcripts (22), and Chimeric LINE Finder (CLIFinder), a bioinformatics tool we developed to identify chimeric reads that correspond to potential LCTs in RNA-seq data (23).

Glioma is the most frequent primary malignant brain tumor. The most aggressive forms, mainly glioblastoma multiform, are molecularly defined by the presence of wild-type isocitrate dehydrogenase 1 and 2 (*IDH*wt) (24,25). *IDH*wt glioma is a major source of morbidity and mortality, because it is almost always fatal. Despite aggressive treatment, these cancers are highly recurrent, leading to a median survival time after diagnosis that does not exceed 18 months. It has been proposed that therapeutic resistance and tumor relapse rely on a subpopulation of cells within the tumor with stem cell characteristics, called glioma stem cells (GSC) (26,27).

In the present study, we used CLIFinder to explore the LCT landscape in *IDH*wt gliomas and GSC cell lines to evaluate whether they warrant future investigations as potential players in aggressive glioma biology. We found that LCTs are expressed in healthy brain and that their transcriptional activity/deregulation in glioma follows that of their host locus.

## Results

### Most chimeras including L1PA1 to L1PA7 elements correspond to LCTs

**Figure 1:**
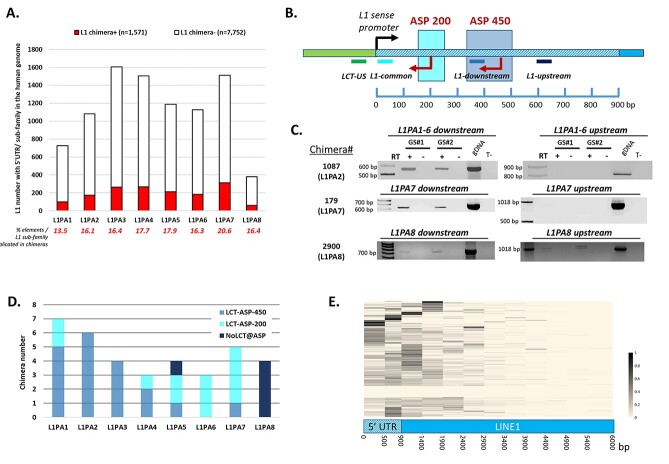
LCTs are produced from the recent L1PA1 to L1PA7 L1 subfamilies. (**A**) Percentage of L1 elements associated with CLIFinder-detected chimeras (red) among all L1 elements with a 5′ UTR region and belonging to the human L1PA1 to L1PA8 subfamilies. (**B**) RT-PCR-based 5′ L1 walking approach. This technique combines a LCT-specific primer (LCT-US, green bar), located in the unique genomic sequence (in green), and primers (grey lines) located either downstream or upstream of the L1-ASP 200 and 450 bp regions (highlighted boxes) in the L1 5′ UTR sequence (in blue). (**C**) Examples of RT-PCR 5′ L1 walking results in two glioma samples (GS#1 and #2). The chimeras Id_1087 (L1PA2 subfamily) and Id_179 (L1PA7 subfamily) correspond to LCTs with a transcription start site (TSS) in the L1-ASP-450 region. The chimera Id_2900 (L1PA8 subfamily) initiates upstream the two L1-ASP regions and therefore, it is not considered to be an LCT. (**D**) Results obtained for the 36 tested chimeras distributed according to the L1 subfamilies. The TSS of each chimera is defined according to the PCR results obtained with the L1 downstream and upstream primers. Chimeras are defined as LCTs when no amplification was obtained with the L1 upstream primer on the tumor cDNA samples. (**E**) The long-read SMRT sequencing dataset was used to generate a heatmap showing TSS occurrence along the full-length L1 element associated to the 226 putative LCTs defined by CLIFinderin GSCs. Twenty-two LCTs were not detected in the long-read SMRT sequencing dataset and are shown as white horizontal lines. Among the other 204 LCTs, 84.80% had at least one read with a TSS included in the 0-500 bp 5′ UTR region, and this percentage increased to 98% when considering TSS in the whole 5′ UTR.

First, we generated Ribo-Zero stranded paired-end RNA-sequencing (RNA-seq) data to analyze the whole transcriptome (total RNA) of three healthy brain and 8 *IDH*wt glioma samples with CLIFinder. As the L1-ASP is active in the more recent subfamilies (5,7,19), we used this approach to identify chimeric transcripts involving one of the 9123 elements with a 5′ UTR from the L1PA1 to L1PA8 subfamilies (Supplementary Material, Table S1). Although we obtained 90 M of reads, each RNA-seq dataset covered only partially the whole LCT transcriptome of that sample. Accordingly, the number of detected LCTs increased with the number of samples analyzed, indicating that for extensive genome-wide LCT identification sequencing data from several samples must be pooled (Supplementary Material, Fig. S1). Finally, by pooling the RNA-seq data from all 11 samples, we could identify a total of 1571 chimeras (Supplementary Material, Table S2), and observed that all eight subfamilies were involved in their production. The percentage of L1 elements implicated in chimera production ranged from 13.5% for L1PA1 to 20.6% for L1PA7 (Fig. 1A).

LCT transcription initiation from L1-ASP was initially mapped between positions +300 and +500 of the L1PA1 5′ UTR (4), but can also occur between position +150 and +250 (7,20). Chimeras detected with our approach may initiate from one of these two regions, or may also correspond to larger transcripts that encompass the L1 5′ UTR in antisense orientation. Therefore, we next evaluated the proportion of chimeras that corresponded to LCTs (i.e*.* they initiated from one of these two L1-ASP regions). To this aim, we designed a RT-PCR 5′ L1 walking approach using three primers, located at different positions of the L1 5′ UTR, and one LCT-specific primer (LCT-Unique Sequence = LCT-US), located in the unique adjacent sequence, to delineate the area where each chimera initiated (i.e. +200 or +450 in the 5′ UTR or upstream) (Fig. 1B).

We tested 36 chimeras (7 from L1PA1, 6 from L1PA2, 4 from L1PA3, 3 from L1PA4, 4 from L1PA5, 3 from L1PA6, 5 from L1PA7, and 4 from L1PA8) in two glioma samples (GS#1 and #2). We could amplify 19 of them using the two primers located in the first 500 bp of the 5′ UTR, but not with the “upstream” primer, for instance chimera 1087 and 179 (Fig. 1B–D). This pattern was representative of a transcription start site (TSS) located in the classical +450 L1-ASP region. For 12 chimeras, we obtained an amplification product with the most ‘downstream’ L1 primer (called L1-common), but not when using the L1-downstream and L1-upstream primers, flanking the +450 ASP position (Fig. 1B–D). This suggests that these chimeras might initiate in the +200 L1-ASP region. We could amplify the last five chimeras (including the four L1PA8 chimeras tested), such as chimera 2900, using the three primers, indicating they do not initiate from the 5′ UTR L1-ASP regions, but from an undetermined upstream region (NO_LCTs). This analysis confirmed that the four tested L1PA8 chimeras corresponded to larger transcripts, and that 31 of the other 32 chimeras (96.8%), which were associated with L1 elements that belonged to the L1PA1–L1PA7 subfamilies, were LCTs the transcription of which started at one of the two already described L1-ASP regions.

To evaluate whether this observation can apply to a whole population of chimera, we also analyzed by Ribo-Zero RNA-seq two *IDH*wt GSC lines (GSC1 and GSC2). By pooling the data of these two samples, we could identify by CLIFinder 226 chimeras that involved L1 elements from the L1PA1 to L1PA7 subfamilies. Then, to determine the proportion of chimeras that corresponded to LCTs, we mapped their TSS along the L1 sequence using a long-read single molecule real-time (SMRT) sequencing dataset generated from three GSC lines (GSC1, GSC2 and GSC6). Among the 226 chimeras identified by CLIFinder, 22 were not detected in this dataset. The remaining 204 had at least one TSS within the L1 sequence, including the L1 coding region. Nevertheless, we observed a TSS hotspot within the 900 bp of the L1 5′ UTR (Fig. 1E). Specifically, 98% (201/204) of these chimeras had one or more 5′ ends in the L1 5′ UTR and 85% (173/204) within the first 500 bp region that contains the two described ASP regions. This original observation highlights that besides initiation from the already described L1-ASP region in the 5′ UTR, some LCTs can also have secondary initiation site(s) within the L1 coding sequence, and older L1 elements may be involved in LCT transcription.

These findings suggest that the majority of the 1509 chimeras implicating these seven subfamilies (L1PA1 to L1PA7) may correspond to LCTs that initiate at the L1-ASP. For the rest of this study, we will consider only L1PA1 to L1PA7 as recent L1 subfamilies.

Previous studies based on EST querying showed that among the 8744 recent L1 elements with a 5′ UTR sequence from the L1PA1 to L1PA7 subfamilies, 161 (1.9%) are associated with LCT transcription in various human normal tissue and cancer types and cell lines (4,7,14,17,18,20). By comparing the genomic positions of the 1509 LCT-producing L1 elements identified by CLIFinder and of these 161 “LCT-EST” loci, we determined that 65 L1 elements corresponded to already known LCT-EST loci (Supplementary Material, Fig. S2) among which 10 were previously described as producing LCT-ESTs in normal and malignant brain samples ((7); Supplementary Material, Fig. S3).

In conclusion, in normal brain and glioma samples, our approach identified 40% of the 161 L1 elements previously associated with LCT-EST production. It also identified 1444 new recent L1 elements implicated in LCT transcription, thus implicating 17.25% of human L1PA1 to L1PA7 retrotransposons (with a 5′ UTR) in LCT expression in *IDH*wt glioma and normal brain.

### Most LCTs are unspliced at their 5′ end and can be polyA and non-polyA transcripts

The size of the 1509 LCTs ranged from 132 to 45 574 bp. The median size of the LCTs identified by only 1 read (*n* = 453) was 306 bp, and 77% of them were smaller than 400 bp. As the L1 antisense 5′ UTR contains two donor splicing sites, we evaluated the splicing status of these transcripts by analyzing three parameters: the genomic distance between the L1 side and the unique sequence for each chimera, the total chimera size, and the position of the splice junctions identified by TopHat around the L1 first nucleotide in the 11 samples (Supplementary Material, Fig. S4). We found evidence of splicing for 44 LCTs (including for the 17 spliced LCT-ESTs already described). This indicated that most LCTs identified by CLIFinder resulted from continuous transcription from the L1-ASP to the unique adjacent sequence and that only 2.9% of LCTs were spliced at their 5′ end.

**Figure 2:**
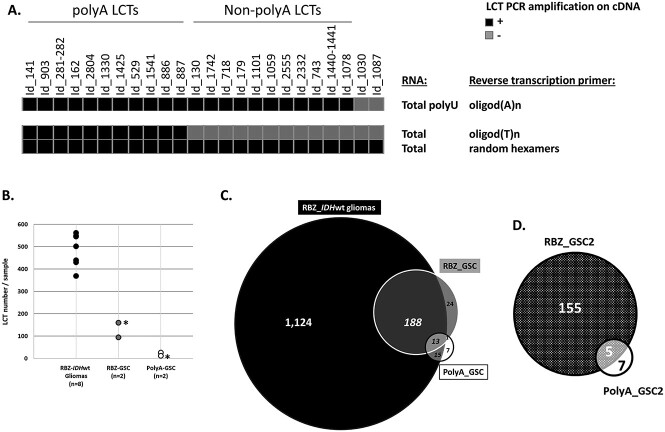
LCTs can be polyA and non-polyA transcripts. (**A**) Heatmap describing the PCR amplification results for 24 LCTs using glioma cDNA obtained with different reverse transcription conditions (see Methods). PCR amplifications were performed using a LCT-specific primer (LCT-US) and the L1 common primer (see Fig. 1B). (**B**) Comparison of the number of LCTs (identified by CLIFinder) for each sample (glioma samples and GSC lines) and according to the RNA-seq approach used (Ribo-Zero (RBZ) and PolyA) ^*^: This GSC2 cell line was analyzed by PolyA- and RBZ-RNA-seq. (**C**) Comparison of the number of LCTs identified by CLIFinder in three datasets: PolyA RNA-seq dataset of two GSC cell lines (GSC2 and GSC6), Ribo-Zero RNA-seq dataset of two GSC lines (GSC2 and GSC1), and Ribo-Zero RNA-seq dataset of eight *IDH*wt glioma samples. (**D**) Comparison of the number of LCTs (identified by CLIFinder) associated with L1PA1 to L1PA7 retrotransposons in the RBZ and in PolyA RNA-seq datasets of the GSC2 line (data obtained using the same RNA sample).

Our strategy, based on Ribo-Zero RNA-seq, identifies LCTs regardless of their polyadenylation (polyA) status. To determine whether LCTs were polyA transcripts, we tested the presence of 24 LCTs, selected among the 31 we previously validated, in cDNA obtained by reverse transcription of total RNA from two glioma samples (GS#1 and #2) with random hexamers or with an oligodT oligonucleotide. We could amplify all 24 LCTs in cDNA obtained with random hexamers, but only 11 in cDNA reverse transcribed with the oligodT primer (Fig. 2A). The absence of amplification for 13 LCTs could be due to the presence of a non-polyA transcript or by inefficient full-length reverse transcription (e.g*.* long transcript). Therefore, we incubated the same RNA samples with a poly(U) polymerase to add an artificial polyU tail to their 3′ end, before the reverse transcription step performed using an oligodA oligonucleotide. In this case, we could amplify 22 of the 24 LCTs (Fig. 2A), suggesting that at least 11, and up to 13 of the 24 studied LCTs were not polyadenylated.

In a more systematic approach, we compared the data obtained by Ribo-Zero RNA-seq of GSC1 and GSC2 samples with those obtained by polyA RNA-seq of GSC2 and GSC6 samples. The number of LCTs detected per sample was always higher in the Ribo-Zero RNA-seq datasets (Fig. 2B). Similarly, the total number of LCTs identified was significantly higher in the two GSC samples analyzed by Ribo-Zero RNA-seq than in the two samples analyzed by PolyA RNA-seq (*n* = 226 versus *n* = 36) (Fig. 2C). Specifically, for the GSC2 sample (analyzed using both RNA-seq approaches), CLIFinder identified 160 and 12 LCTs associated with recent L1 retrotransposons in the Ribo-Zero and PolyA datasets, respectively, among which five were shared (Fig. 2D). The 13-fold increase in LCTs detected in the Ribo-Zero datasets is not explained only by the 2-fold higher sequencing depth (90 M versus 40 M of reads), and supports the hypothesis that a relevant LCT subset is not polyadenylated.

Importantly, most LCTs detected in the GSC samples by both RNA-seq approaches were also detected in the pooled RNA-seq data from the eight *IDH*wt glioma samples (Fig. 2C), highlighting that LCTs are recurrently expressed in glioma samples from different patients.

### LTC-producing L1 retrotransposons in brain and *IDW*wt glioma samples tend to localize in genes with brain-related functions

Compared with the 8744 recent L1 retrotransposons possessing a 5′ UTR, the 1509 L1 retrotransposons involved in LCT production did not show a specific size (*n* = 1115, 73.9%, were 6 kb in length; data not shown) or genomic distribution (Supplementary Material, Fig. S5).

**Figure 3:**
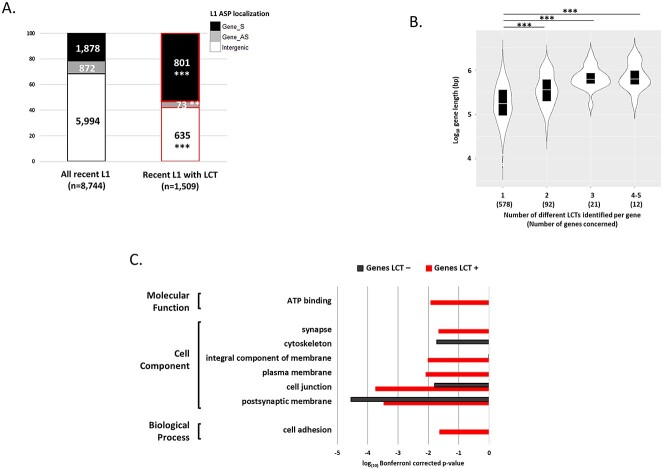
Characteristics of the 1509 LCTs identified by CLIFinder in *IDH*wt glioma and normal brain samples. (**A**) Comparison of the localization, relative to annotated genes, of all recent L1 with a 5′ UTR (*n* = 8744) and of recent L1 retrotransposons associated with the 1509 LCTs. The L1-ASP orientation is given relative to the gene orientation. The significant enrichment and depletion of intragenic and intergenic LCT-producing L1 retrotransposons, respectively, compared with all recent L1 retrotransposons, was confirmed with the binomial test (^*^^*^^*^P < 0.001). (**B**) Number of genes in which the indicated number of LCTs was identified by CLIFinder; ^*^^*^^*^P < 0.005 (one way-ANOVA and Mann–Whitney post hoc test). (**C**) Gene Ontology terms (GOTERM) enriched in genes containing recent L1 retrotransposons associated with LCTs (LCT+, red) compared with genes containing recent L1 retrotransposons that do not produce LCTs (LCT−, black). For each GOTERM category, the most significant terms (Bonferroni corrected *P* < 0.05) are shown.

Conversely, 58% of them (874/1509) were intragenic, compared with 31.5% for all recent L1 in the human genome (Fig. 3A). Surprisingly, only 8% of intragenic L1-ASPs producing LCTs were in the antisense orientation relative to their host gene, compared with 32% for the whole L1 population considered here. Most host genes (*n* = 578) contained only 1 LCT-producing L1, 92 genes contained 2 LCT-producing L1, and 33 genes contained 3–5 LCT-producing L1 retrotransposons. The latter genes were large genes with a size ranging from 0.16 to 2.3 Mb (Fig. 3B). Gene Ontology analysis showed that the host genes of the 874 intragenic LCT-producing L1 were significantly enriched in genes involved in brain function, i.e*.* synapse function and components (Fig. 3C). The host genes of recent L1 elements that do not produce LCTs in brain samples were enriched in genes with more ubiquitous functions, such as cytoskeleton components.

### LCTs are expressed in healthy brain and aggressive glioma

**Figure 4:**
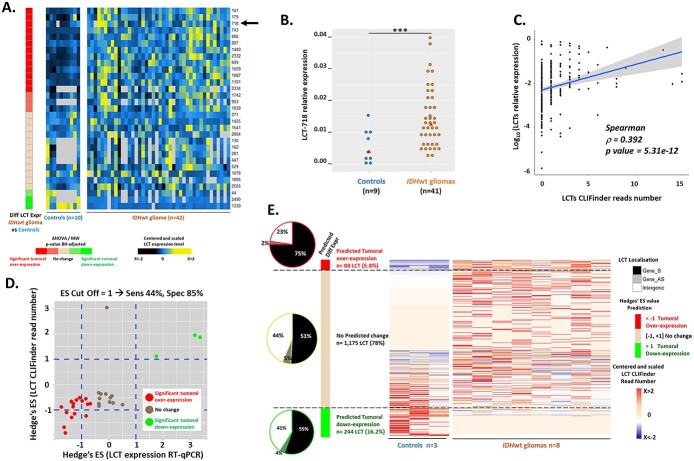
LCTs are expressed in normal brain and glioma samples, but with different expression levels for a LCT subset. (**A**) Heatmap showing the centered-scaled expression levels between samples for each LCT and the differential expression of the 31 validated LCTs in *IDH*wt glioma and control samples (Mann–Whitney, MW, test followed by the Bonferroni-Holm, BH, correction). Significant LCT expression up- or down-regulation in glioma samples is represented by red and green squares, respectively. (**B**) LCT_718 (shown by an arrow in (A) is an example of LCT significantly overexpressed in glioma samples. (^*^^*^^*^ BH adjusted *P* < 0.005). (**C**) Positive Spearman’s correlation between the CLIFinder reads number for the 31 validated LCTs and their relative expression (log_10_) measured by RT-qPCR in two control and eight *IDH*wt glioma samples. (**D**) Hedges’ ES determination. For each of the 31 validated LCTs, the Hedges’s ES (*g* value) between *IDH*wt glioma and control samples was calculated for the CLIFinder reads number and RT-qPCR relative expression. An ES cut-off value of 1 for the CLIFinder reads number was determined with a sensitivity of 44% and a specificity of 85%. (**E**) Heatmap representations of the centered-scaled LCT CLIFinder reads number and of the predicted differential expression in *IDH*wt glioma versus control samples for the 1509 LCTs. Hedges’ *g* values <−1 and >+1 predict a significant up- (red) and down-regulation (green), respectively. Values between these cut-offs predict no expression change (beige). For each expression group (up- and down-regulation), the localization of the LCT-producing L1 element, relative to the annotated genes, is shown in the pie charts: intragenic LCTs transcribed in the sense of the gene (Gene-S) in black, intragenic LCTs transcribed in the opposite sense (Gene_AS) in gray, and intergenic LCTs in white.

As previous studies suggested that some LCTs could be tumor-specific (7,19,20), we asked whether LCT expression differed in glioma and healthy brain samples. As shown before (Supplementary Material, Fig. S1), extensive genome-wide LCT identification required to pool data of several samples. In agreement, CLIFinder analysis of the RNA-seq data from three healthy brain and eight *IDH*wt glioma samples did not allow assessing the LCT transcriptional status in these two tissue types. Therefore, we first quantified, by RT-PCR, the expression of the 31 validated LCTs in 10 brain control and 41 *IDH*wt glioma samples. All 31 LCTs were expressed in both control and glioma samples (Fig. 4A), although at different levels. Specifically, 16 were upregulated (e.g*.* LCT 718; Fig. 4B) and 3 were downregulated in glioma samples (Fig. 4A).

Moreover, the relative expression, measured by RT-qPCR, of the 31 validated LCTs was positively correlated with their reads number determined by CLIFinder (Spearman’s Rho = 0.395, *P* < 2.2e-16) (Fig. 4C). This suggests that the CLIFinder reads number represents a semi-quantitative value than can be used to predict the transcriptional status of the 1509 LCTs in brain and tumor samples. Therefore, we calculated the Hedges’ *g* effect size (Hedges’ ES) (28). To establish a Hedge’s *g* cut-off value that represented the best compromise between sensitivity and specificity, we compared the *g* values of the 31 validated LCTs, obtained using their RNA-seq reads number, with those obtained using their relative expression by RT-qPCR and annotated according to their differential expression in tumor samples compared with controls (Fig. 4D). This allowed us to define a Hedges’ *g* cut-off value of ±1 (44% of sensitivity and 85% of specificity) (Fig. 4D).

Using this cut-off value, the Hedges’ *g* values obtained for the 1509 LCTs predicted that 244 (16.2%) and 88 (5.8%) of them were down- and up-regulated, respectively, in *IDHwt* glioma versus control samples (*g* values: >1 and <−1, respectively) (Fig. 4E). Note that the ‘upregulated’ category does not distinguish between LCTs that are overexpressed and LCTs that are expressed only in glioma samples.

Altogether, these observations indicated that most LCTs are not glioma-specific. They suggested that most LCTs are produced in healthy brain and also aggressive glioma, and that ~20% of them shows tumor-specific down- or up-regulation.

### Aberrant DNA methylation and genome copy number variations are not the main cause of LCT expression deregulation in glioma samples

**Figure 5:**
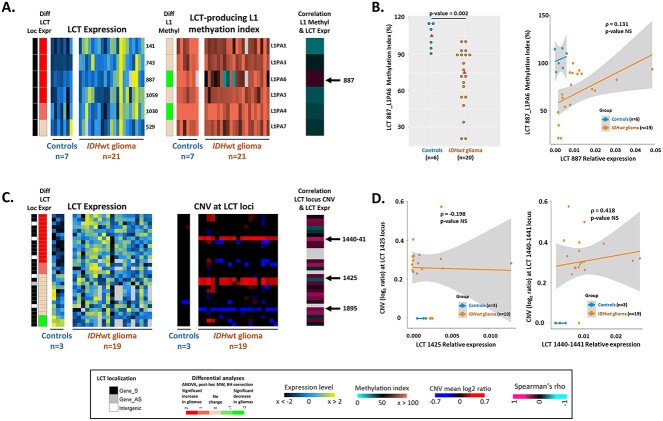
L1 hypomethylation and CNVs are not the driving force of LCT expression changes in *IDH*wt glioma samples. (**A**) Heatmaps summarizing the LCT expresseion and methylation index of the L1 5′ UTR that produce the six indicated LCTs. The correlation analysis between these parameters is shown on the right panel. (**B**) Detailed results obtained for LCT_887 (black arrow in A), in control and glioma samples. Although the L1 promoter methylation index is significantly lower in *IDH*wt glioma than in control samples, the Spearman’s correlation analysis did not highlight any negative correlation between L1 promoter methylation index and LCT_887 expression level. (**C**) Heatmaps summarizing LCT expression and CNV data for the 31 validated LCT loci. The results of the Spearman’s correlation analysis are shown in the right panel. (**D**) Example of results obtained for the LCTs_1425 and_1440–1441. These two LCTs are on chromosome 7 that is duplicated in *IDH*wt glioma; however, the expression level of LCT_1425 was not changed in glioma compared with control samples, and no correlation was detected between LCT_1440–1441 expression level and CNV.

To elucidate the causes of the tumor-specific upregulation of some LCTs, we first asked whether it could be explained by L1 promoter hypomethylation, resulting from the global hypomethylation of tumor DNA, as previously demonstrated for the *L1-MET* LCT in cancer cell lines (17). To test this hypothesis, we optimized the Quantitative analysis of DNA methylation by qPCR (qAMP) technique (29) to assess the promoter methylation level in 6 of the 31 validated LCT-producing L1 elements. Five of the associated LCTs were overexpressed in glioma samples, while one displayed the same expression level in control and glioma samples. The qAMP approach, based on the use of methylation-sensitive and methylation-dependent restriction enzymes followed by real-time PCR, allows the analysis of several CpG sites localized in the immediate 5′ L1 sequence, thus covering the CpG sites previously described as informative (17) (Supplementary Material, Fig. S6A–C). Comparison of the methylation index obtained for each of the six loci in 7 brain controls and 21 *IDH*wt glioma samples identified two L1 elements associated with LCTs overexpressed in tumors and in which promoter methylation was reduced in the *IDH*wt samples compared with controls (−28% for LCT-1030, *P* = 0.001; and −30% for LCT-887, *P* = 0.002) (Fig. 5A and B; Supplementary Material, Fig. S6C). However, we did not detect any significant negative correlation between LCT relative expression and promoter methylation of the associated L1 element at these two hypomethylated loci nor at the other four. Moreover, the methylation index for the six loci remained high in most glioma samples.

Next, to determine whether genomic rearrangements, which are often observed in cancer cells, could contribute to LCT deregulation, we analyzed copy number variation (CNV) in the genomic loci of the 31 validated LCTs in 3 controls and 19 *IDH*wt samples. *IDH*wt samples are characterized by chromosome 7 gain and chromosome 10 loss (24). Accordingly, most *IDH*wt glioma samples carried extra and fewer copies of the L1 elements located on chromosome 7 and 10, respectively (Fig. 5C). However, there was no obvious link between CNV in glioma samples and LCT expression variation. Indeed, among the three LCT-producing L1 elements on chromosome 7 (LCT_1425, LCT_1440-41 and LCT_1451), only LCT_1440-41 was overexpressed in glioma samples. Moreover, expression of the chromosome 10-associated LCT_1895 was unchanged in glioma samples relative to controls, although the L1 copy number was decreased in glioma samples. Finally, the 15 loci associated with the other overexpressed LCTs did not show any CNV. Moreover, comparison of CNV and expression data at each LCT in each sample did not highlight any significant correlation between CNV and LCT expression (Fig. 5C and D).

Combined, these observations indicated that aberrant DNA methylation and genome CNV might contribute, but are not the main cause of LCT deregulation in glioma samples.

### LCT transcription from the L1-ASP occurs in transcriptionally active loci

**Figure 6:**
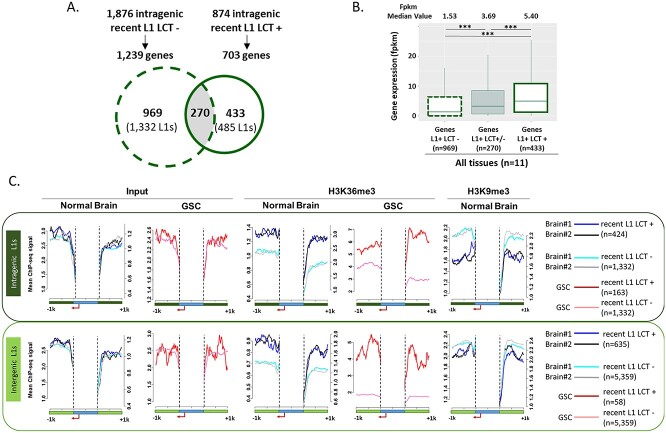
Recent LCT-producing L1 retrotransposons are localized in transcriptionally active regions. (**A**) Comparison of genes containing a recent L1 retrotransposon associated or not with the transcription of a LCT in this study. 270 genes contain at least two recent L1 retrotransposons with divergent LCT expression status. **(B**) Comparison of the expression levels of genes containing a recent L1 retrotransposon that expresses (Genes L1+ LCT+, *n* = 433) or not (Genes L1+ LCT−, *n* = 969) LCTs. The box plots lower and upper limits indicate the 25th and 75th percentile, respectively, and the middle line represents the median. The median fpkm numeric value is given above each group; ^*^^*^^*^P < 2.2e-16 (*T*-test). (**C**) Analysis of ChIP-seq reads density data for input, H3K36me3 and H3K9me3 for two normal brain samples and one GSC cell line. Plots are centered on a ±1 kb window according to the L1 start and end (blue horizontal bar with the L1-ASP in red). Both intragenic (dark green) and intergenic (light green) L1 retrotransposons are considered. Loci retained as recent L1 retrotransposons not associated with LCT transcription (dashed lines) correspond to L1 elements never associated with LCT in our study (*n* = 1332 intragenic and *n* = 5359 intergenic L1). For analysis of normal brain samples, all recent L1 retrotransposons associated with CLIFinder-detected LCTs in controls and *IDHwt* gliomas were retained (*n* = 424 intragenic L1 and *n* = 635 intergenic L1 retrotransposons). For analysis of the GSC sample, only recent L1 retrotransposons associated with LCTs identified by CLIFinder in GSC samples after Ribo-Zero RNA-seq were analyzed (*n* = 232 intragenic L1 and *n* = 58 intergenic L1 retrotransposons). Plots represent the mean ChIP-seq signal values for recent L1 retrotransposons associated with LCT transcription (dark colors) and for recent L1 retrotransposons not associated with LCT transcription (bright colors).

The previous data argue against a central role for DNA methylation in LCT production regulation. Specifically, the observation that LCT-producing L1 elements in control and *IDH*wt glioma samples tend to localize in genes with brain functions suggests that the L1-ASP activity of recent L1 could be influenced by the host locus transcriptional activity, as observed for the sense promoter (30,31). To test this hypothesis, we first compared the expression level of genes that contain one or several LCT-producing L1 retrotransposons (*n* = 433 genes/485 L1) and of genes hosting one or several recent L1 not associated with LCT production (*n* = 969 genes/1332 L1) (Fig. 6A). Comparison of the RNA-seq data from normal brain (*n* = 3) and *IDH*wt glioma (*n* = 8) samples indicated that the expression level of genes containing LCT-producing L1 retrotransposons was significantly higher (Fig. 6B). This was true when the analysis included all samples (control and glioma) (Fig. 6B), and also when focused only on controls or aggressive glioma samples (Supplementary Material, Fig. S7A). Thus, intragenic LCT transcription from the ASP for recent L1 retrotransposons seems to occur preferentially from transcriptionally active genes.

In agreement, analysis of chromatin data of the −1 kb/+1 kb regions surrounding recent L1 retrotransposons from two normal brain samples and one GSC line highlighted that the genomic regions surrounding LCT-producing L1 elements were enriched in histone marks associated with transcribed regions (i.e*.* H3K36me3) compared with those of recent L1 elements not associated with LCT production (Fig. 6C, and Supplementary Material, Fig. S7B). Conversely, the repressive H3K9me3 mark was enriched around regions of recent L1 retrotransposons not associated with LCT transcription (Fig. 6C). We obtained similar results for intergenic L1 retrotransposons, suggesting that those LCT-producing L1 elements are localized in transcribed intergenic regions (Fig. 6C).

Altogether, these observations support the hypothesis that LCT transcription from inter- and intra-genic L1 retrotransposons in normal brain and *IDH*wt glioma samples is associated with the active transcriptional state of the host locus. Like for the L1 sense promoter, the transcriptional activity of the L1-ASP might be influenced by its immediate surrounding genomic sequence.

### LCT expression deregulation in tumors follows that of their host locus

**Figure 7:**
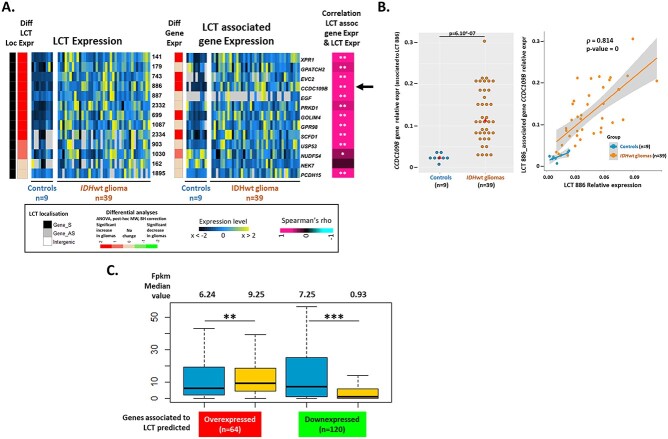
LCT deregulation in glioma samples is linked to transcriptional changes in the host gene. (**A**) Heatmaps summarizing the expression levels quantified by RT-qPCR of the indicated LCTs (13 intragenic LCTs, including 11 overexpressed in *IDH*wt glioma samples) and of their host genes. The host gene expression levels were compared between Controls and *IDH*wt glioma samples and correlated with the LCT relative expression level. Significance of Spearman correlations were assessed by Bonferroni corrected *P*-values and indicated as ^*^P < 0.05, ^*^^*^P < 0.01. (**B**) Example of results obtained for LCT_886 and its host gene *CCDC109B*. LCT_886 is overexpressed in *IDH*wt glioma samples. *CCDC109B* also is significantly overexpressed in *IDH*wt glioma samples compared with controls, and LCT_886 and *CCDC109B* expression are positively correlated (Spearman’s rho = 0.814; *P*-value = 0). (**C**) Comparison of the expression level (fpkm) in control (*n* = 3) and *IDH*wt glioma samples (*n* = 8) of the genes associated with the 64 and 120 intragenic LCTs predicted to be up- or down-regulated, respectively, in tumors by the Hedges’ ES prediction model (Fig. 4E).

Then, to determine whether the tumor-associated change in the expression of some LCTs could be explained by transcriptional deregulation of the host locus, we assessed by RT-qPCR the expression of 13 genes hosting 13 validated LCTs (11 overexpressed and 2 unchanged in tumors) in 9 controls and 39 *IDH*wt glioma samples (Fig. 7A). Six genes were significantly overexpressed in tumor samples (Fig. 7A and B). Moreover, correlation analyses indicated that for all host loci with overexpressed LCTs, the host gene expression was highest in samples with the highest LCT expression (Fig. 7A and B). Univariate linear regression analysis validated these correlations, and multivariate linear regression analysis confirmed the strong positive correlation between the expression of the LCT and associated gene, independently of the sample group.

We next asked whether this observation could be applied to all intragenic LCTs. Therefore, we compared the expression (in fpkm, from RNA-seq data) of genes associated with the 64 and 120 intragenic LCTs predicted to be up- and down-regulated in gliomas, respectively, in controls and *IDH*wt glioma samples. The gene expression in tumor and control samples was similar to that of their associated LCTs (Fig. 7C). Genes associated with upregulated LCTs also were significantly overexpressed in *IDH*wt glioma samples, whereas genes associated with downregulated LCTs also were significantly downregulated.

This suggests that LCT down- and up-regulation in glioma imply a similar transcriptional deregulation of the relevant host locus.

## Discussion

Our study showed that LCT transcription from the ASP region in the L1 5′ UTR is widespread in aggressive glioma, and that 17.25% (*n* = 1509) of recent L1 retrotransposons are involved in their production. It also revealed that these events are not tumor-specific, because LCT transcription occurred also in healthy brain, as previously shown in normal colon (32).

To date, *in silico* and molecular analyses in various human normal tissue and cancer samples and cell lines identified 161 recent LCT-producing L1 retrotransposons (4,7,14,17,18,20). Here, by analyzing healthy brain and *IDH*wt glioma samples using Ribo-Zero RNA-seq and a dedicated bioinformatics tool, CLIFinder (23), we extended the LCT landscape by revealing that more than 1500 recent L1 retrotransposons can produce LCTs in both sample types. We also identified two LCT features that mainly explain this marked increase in the ability to detect LCTs: most are unspliced at their 5′ end, and they can be polyA or non-poly-A. Unlike previous approaches (4,7,14,17,18,20), our strategy is not biased toward spliced transcripts and uses total RNA. Therefore, this strategy requires RNA-seq data with high reading depth and/or the pooling of data from several samples to be representative. The vast majority of LCTs we identified are unspliced at their 5′ end, although two splicing donor sites are located in the L1 promoter. This finding was obtained using RNA-seq data based on total RNA and also polyA RNA, arguing against a technical bias linked to nascent RNA sequencing, and stresses that continuous transcription from the L1-ASP to the adjacent unique sequence is a major feature of the LCT 5′ end. However, this does not preclude the possibility of downstream splicing events in the LCT transcripts, as observed in most of the previously characterized LCTs. Moreover, 89% of the LCTs we identified in GSC lines are also found in glioma samples isolated from different patients, demonstrating that our approach allows revealing a LCT tumor-type specific signature. Altogether, these observations validate our approach as relevant to investigate genome-wide LCT expression from L1-ASP in a tissue type. It should be noted that due to the CLIFinder settings used here, our conclusions are valid only for LCTs that initiate at ASP regions located in the first 500 bp of the L1 elements. However, our analysis of long-read sequencing datasets revealed that LCTs may also initiate in the coding sequence of L1. To better characterize these LCTs and to determine whether they may have arisen from older subfamilies, such as L1PA8, they should be compared with a reference set of L1 coding sequences (i.e. 900–1900 bp). The tunable nature of CLIFinder allows performing other refined analysis. For instance, it can be used to identify also a tumor sample-specific, rather than tumor type-specific signature. Such tumor type-specific analyses can be necessary for taking into account potential tumor-related genome rearrangements and the presence of additional L1 copies not present in the reference genome. In this case, the tumor genome, if available, should be used for the CLIFinder alignment step, and not the human reference genome.

**Figure 8:**
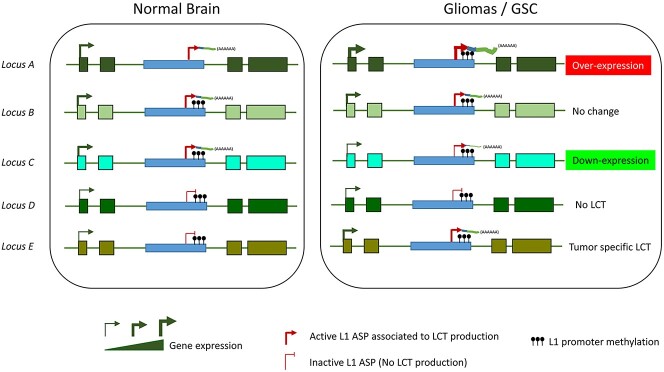
Working model of LCT transcriptional regulation in normal brain and in glioma/GSCs. Our study supports a model whereby the transcriptional activity of the host genomic locus is the main driving force of LCT expression. We propose that this model is applicable particularly when the host gene and the L1-ASP are in the same orientation. Accordingly, in normal brain, LCTs are widely expressed. Changes in the host gene/genomic locus expression level in glioma and GSCs will lead to similar changes in LCT expression level, regardless of the L1 promoter methylation status. Note that according to this model, a gene specifically expressed in glioma can contribute to the expression of a glioma-specific LCT (locus E).

A key finding of our study is that the L1-ASP transcriptional activity and that of the host locus are coupled (Fig. 8). This observation provides an experimental evidence to the model proposed by Nigumann *et al.* (14) about two decades ago according to which L1-ASP activation ‘*could be a consequence of the activation or chromatin opening of the cellular host genes.*’ In agreement, we observed that in normal brain and in GSC lines, LCT-producing L1 retrotransposons are embedded in genomic regions enriched in H3K36me3, a chromatin mark associated with transcription elongation. As intergenic LCT-producing L1 retrotransposons present a similar feature, we propose that transcription events in general, regardless of their occurrence in intragenic or intergenic regions, can influence the L1-ASP promoter activity. This is similar to what described for intragenic L1 sense promoter activation that is restricted to the cells in which the host gene is transcribed (30,31), indicating that the host locus transcriptional activity influences the whole L1 5′ UTR transcriptional activity. However, as sense and antisense L1 promoter activities generally do not co-exist on the same L1 sequence (6,31), other regulation levels are likely to refine transcription from the sense and antisense promoters, particularly the relative orientation of the L1 element and the host genes. Indeed, 68% of all intragenic recent L1 retrotransposons are in the opposite orientation as their host gene, and this percentage increases to 92% for LCT-producing L1 retrotransposons in brain and glioma samples. This suggests that sense-oriented progression of the transcriptional complex through the L1-ASP (or L1 sense promoter) is required to promote the transcriptional activity and/or the stability of the resulting LCT (or L1) transcript. This model, the underlying molecular bases of which remains to be determined, provides a mechanism whereby L1 sense and antisense promoter activities are mutually exclusive. It also ensures that intragenic L1 retrotransposons are not a source of antisense transcripts relative to those of the host gene that could affect the locus epigenetic regulation and mRNA stability through the formation of double-stranded RNA. Our study also bring some insights into the role of DNA methylation in the control of L1-ASP activity. In line with a comprehensive study on two L1-ASPs in various cancer cell lines (33), but unlike what described for the L1-*MET* LCT (17), we did not observe any inverse correlation between L1 promoter methylation and L1-ASP activity (evaluated by measuring the relative LCT expression level). It must be noted that in all studies on this question (17,33), including the present work, DNA methylation was assessed at CpG sites located in a CpG island that overlaps with the internal L1 sense promoter region, but downstream of the L1-ASP transcription starting site. Therefore, the role of this CpG island in the control of the L1-ASP could be questioned. Moreover, the L1-ASP core activity and putative regulatory regions (position +450 to +800 of the 5′ UTR) (4) are depleted in CpG sites, further arguing against a central role of DNA methylation in the control of L1-ASP activity. Altogether, these data suggest that the host gene locus transcriptional activity is the main driving force of LCT expression in physiological and pathological conditions. Therefore, and unlike what observed at the L1 sense promoter, L1-ASP activity is more influenced by the cancer-associated changes in the host gene expression level rather than by the cancer-associated disruption of the DNA methylation pattern. This leads to the question of whether and how LCTs play a functional role during tumorigenesis. In a simple model, tumor-specific LCTs, hosted by genes expressed in the tumor, might influence the expression of their host and/or surrounding genes, thus directly contributing to tumorigenesis. However, in aggressive glioma, this model is not valid for the majority of LCTs because they are also expressed in healthy brain. We could hypothesize that changes in LCT expression at a given locus might modify its role in cancer cells compared with normal cells. *L1-MET* and LCT13, the two main examples of LCT role in tumorigenesis, are both expressed in healthy cells. Their ability to promote oncogenic functions, as proposed for *L1-MET* (17), or to affect the expression pattern of surrounding genes, as proposed for LCT13 (19), is thought to be related to a change of their expression level in cancer cells (17,19). Another open question is whether LCT expression level changes can modify their length and splicing signature in tumor cells. Future studies should focus on this key question and on evaluating the coding potential of these LCT transcripts. Within this frame, we have initiated a long read sequencing approach to analyze three GSC samples (see Fig. 1E). This yet in development approach requires now to be conducted to more samples and to benefit from the development and validation of dedicated bioinformatics tools to identify full-length LCT sequences in healthy brain and aggressive glioma.

In conclusion, our study describes the full LCT expression profile in brain and glioma samples. Unexpectedly, this analysis highlighted that LCT expression is not restricted to glioma, but is widespread also in normal brain: approximately 17% of all recent L1 retrotransposons with a 5′ UTR, from the L1PA1 to L1PA7 subfamilies, produces a LCT in brain and glioma. Our study also revealed that the transcriptional activity from L1-ASP and from the promoter of the host locus are coupled, suggesting that LCT transcription is mainly regulated by the host locus transcriptional activity and chromatin signature.

## Materials and Methods

### Tumor and control samples

Diffuse glioma samples from adult patients who underwent surgical resection between 2007 and 2014 were obtained from Clermont-Ferrand University Hospital Center, France (‘Tumorothèque Auvergne Gliomes’ ethical approval DC-2012-1584). This study was approved by the relevant ethics committees and competent authorities, and the study protocols follow the World Medical Association Declaration of Helsinki. Written informed consents were provided by all patients. Samples were isolated as previously described (34,35). In this study, the 42 included aggressive glioma samples carried wild type *isocitrate dehydrogenase* (*IDH*) gene (*IDH*wt) (25).

Ten control brain samples (healthy controls; samples removed by autopsy 4–16 h after accidental death) were obtained from the Brain and Tissue Bank of Maryland (mean age of 27.3 years, standard deviation ±2 years). These samples, identified by the Brain and Tissue Bank of Maryland as corpus callosum (*n* = 8) and frontal cortex (*n* = 7), correspond to white matter enriched in astrocytes and oligodendrocytes, and are relevant non-cancer controls for gliomas. Before use, tumor and control samples were homogenized by cryogenic grinding, and each sample was aliquoted in at least three vials for genomic DNA, RNA and chromatin extraction. All samples were stored at −80°C until use.

Cell pellets from three GSC lines (GSC-1, GSC-2 and GSC-6) derived from patients with *IDH*wt gliomas were obtained from Poitiers University Hospital Centre, France, and were previously characterized (36–38).

### List of ASP-containing L1 elements

The coordinates of L1 elements were obtained from the RepeatMasker database deposited in the UCSC Table Browser. Recent L1 elements were selected based on their RepName (L1P1, L1HS, L1PA2, L1PA3, L1PA4, L1PA5, L1PA6, L1PA7, L1PA8). This list was then filtered using repLeft and repStart <400 and repEnd >600 to retain only L1 elements with an ASP. The final list is in Supplementary Material, Table S1.

### Illumina RNA-sequencing and LCT identification using CLIFinder

Total RNA was isolated from frozen tissue samples and frozen cell pellets as previously described (35). Strand-oriented RNA-seq was performed using total RNA (*n* = 3 brain control, *n* = 8 *IDH*wt, and *n* = 2 GSC samples: GSC-1 and GSC-2) and also polyA mRNA from the GSC-6 and GSC-2 samples (34).

CLIFinder was used as previously described (23) to extract chimeras from stranded paired-end RNA-seq data. The analyses were based on the reference annotated human genome GRCh37/hg19. The reference set of L1 sequences used was a text file containing FASTA sequences that corresponded to the first 500 bp of the 9123 L1 elements (L1PA1 to L1PA8) with a 5′ UTR sequence in the human genome. These sequences were extracted from the Repeat Masker database with UCSC tools. As the reference file contains all potential targeted sequences, the parameters used to select chimeric cDNA of potential interest were:

- 5′ end read sequence in which at least 50 bp that matched a L1 reference sequence (1 mismatch was tolerated to take into account single nucleotide polymorphisms that may be present also in L1 sequences).
- The associated 3′ end read sequence should contain at least one unique sequence (i.e*.* not annotated by Repeat Masker) of ≥30 bp.

Finally, a maximum insert size of 50 kb after alignment to genomic DNA was tolerated between the paired-end reads retained (to allow the identification of spliced chimeras).

In the 11 RNA-seq datasets, CLIFinder identified 1677 chimeras associated with the L1 subfamilies L1PA1 to L1PA8 (Supplementary Material, Table S2). Among these chimeras, 37 were transcribed in the same orientation as the associated L1, and 69 were associated with L1 elements without conserved 5′ UTR, therefore resulting in 1571 chimeras that potentially corresponded to LCTs.

### Long-read SMRT sequencing and LCT TSS identification in GSC samples

To sequence full-length polyA and non-polyA LCT transcripts, a modified Iso-seq protocol (PacBio) was used. For each sample, 3 μg of total RNA was poly-uridinylated using a polyU polymerase (New England Biolabs) following the manufacturer’s recommendations. After poly-uridinylated (polyU) RNA purification with the RNeasy kit (Qiagen) and ethanol precipitation, two reverse transcription reactions were performed (each with 1 μg of polyU RNA) using the SMARTer Kit (Takara) in which the oligodT_(30)_ 3′ SMART CDS Primer II A was replaced by the oligodA_(30)_ 3′ SMART CDS Primer II A. The obtained full-length cDNA (1:10 dilution) was then amplified by PCR (14 cycles) using GXL PrimeSTAR DNA polymerase (Takara) in 20 PCR reactions. PCR products were purified and concentrated (in 21 μl) with two rounds of 1× AMPurePB beads (PacBio) treatment. cDNA size fractionation was then performed on 0.75% agarose gels (without ethidium bromide) to individualize three cDNA fractions: [0.3–2 kb], [2–5 kb] and [>5 kb]. Dedicated biotinylated riboprobes, designed against the 5′ 400 bp sequences of the 8744 recent L1 (myBaits, Arbor Biosciences), were used to perform three independent captures of each cDNA fraction according to the supplier’s recommendations (except for the addition of Block C solution = human Cot-1 DNA). Captured cDNAs were then amplified by PCR using Prime StarGXL DNA polymerase (Takara) with 10, 12 and 14 cycles respectively for the <2, 2–5 and >5 kb cDNA fractions. PCR products were pooled and purified using 0.55× AMPurePB beads (PacBio). The three samples were pooled together and handled for SMRTbell template preparation and sequencing (Sequel II, Gentyane platform). The obtained sequences were treated as follows: (1) consensus sequence establishment using the Iso-seq3 CCS algorithm; (2) selection of the full-length cDNA sequences including the SMARter IIA adapter at both their 3′ and 5′ ends using the Iso-seq3 LIMA tool; (3) trimming of the polyT tail at the 3′ end of each sequence (corresponding to the polyU tail added initially) and finally (4) alignment of the obtained sequences to the hg19 reference human genome using the minimap2 aligner. Only good quality (MapQ ≥ 30) aligned reads were retained in a SAM file (specifying chromosome, start and end genomic coordinates, transcription strand, MapQ value). The genomic coordinates of PacBio read alignments were extracted using the bamtobed tools from the bedtools suite V2.30.0 and the SAM file. Using the GenomicRanges R library, these coordinates were filtered to keep only reads that overlapped with the 226 L1-producing LCTs identified by CLIFinder in GSC Illumina RNA-seq (GSC1 & GSC2) data. Only PacBio reads with potential LCT characteristics were selected: reads containing a 5′ sequence antisense to one of the 226 L1 and a unique genome sequence at its 3′ end. To identify the TSS of the PacBio reads within the L1s, the starting position of each read was repositioned using the L1 coordinates as reference. These data were used to determine the number of L1 that were associated with a PacBio read that initiated in the ASP region or the 5′ UTR of the L1 (i.e. position 0–500 and 0–900 of a L1). To visualize the TSS position of all reads inside each of the 226 L1 elements, the 5′ initiation position of all reads was considered to compute the percentage of reads that initiated along each L1.

### Whole gene expression (fpkm) determination

Illumina stranded RNA-seq reads from three controls and eight *IDH*wt glioma samples were used to determine the normalized gene expression (fpkm), as previously described (34). Briefly, reads were mapped to the human genome (hg19) using TopHat2 (version 2.1.0) and a transcript annotation file from GENCODE (Release 19). Gene expression levels were determined with Cuffquant and Cuffnorm from the Cufflinks suite (version 2.2.1).

### Copy number variation analyses

CNV analyses were performed using the Genome-Wide Human CytoScan HD Array (Affymetrix) and 3 controls and 19 *IDH*wt glioma samples, as previously described (34). To determine whether CNV could be correlated with LCT expression level, the mean log2 ratio of the CNV values and the relative expression level for each LCT locus in the same sample were compared using the Spearman’s correlation.

### Data access

Data are available at the NCBI Gene Expression Omnibus (GEO; https://www.ncbi.nlm.nih.gov/geo/) under the following accession numbers: GSE123892 for the stranded total RNA-seq of control samples and *IDH*wt gliomas; GSE161438 for the stranded total RNA-seq of GSCs; GSM4907328 and GSM180209 for the stranded polyA RNA-seq of GSCs; GSE190930 for the PacBio sequencing of GSCs; and GSE123682 and GSE161275 for the Cytoscan HD data.

### Chimera validation

The LCT-Unique Sequence (LCT-US) primers (using the Primer 3 software: http://primer3.ut.ee/) were designed for the 36 chimeras selected according to their detection by CLIFinder using high or low reads number, they tumor-specificity or not, and their localization (intragenic or intergenic). This primer was combined with a L1 common primer (localized at the beginning of the 5′ sequence of L1 elements). Due to sequence homology, a common primer could be designed for the L1PA1 to L1PA7 subfamilies and a specific L1 common primer for the L1PA8 subfamily. Then, chimera detection was validated by RT-PCR using RNA from two glioma samples (GS#1 and #2) already used for RNA-seq. Briefly, 1 μg of total RNA was treated with DNase I (Promega) according to the manufacturer’s recommendations, and then reverse transcribed using Random Hexamers (Invitrogen) and the SuperScript III Reverse Transcriptase Kit (Invitrogen). After dilution (1:5), 2 μl of cDNA solution was used for PCR amplification with the relevant LCT specific primer, LCT-US and L1 common primers. Primer sequences, PCR conditions, and PCR product amplification size are given in Supplementary Material, Table S3. RT-PCR products were analyzed on 2% agarose gel and those of the expected size were cloned using the pGEM-T Easy Cloning Kit (Promega). One recombinant clone per chimera was sequenced to determine whether the obtained cDNA corresponded to the expected sequence.

### RT-PCR-based 5′ L1 walking approach to localize the TSS of the validated chimeras

RNA available in large amounts and of good quality (RIN > 8) from two glioma samples was used for these experiments. Additional L1 primers, downstream and upstream of the L1-ASP +450 location, were designed using the Primer3 software. Due to the high sequence homology displayed by the 5′ UTR sequence of the L1PA1 to L1PA6 subfamilies, the same L1 downstream and upstream primers were used for chimeras associated with L1 elements from these six subfamilies. However, as some mismatches could impair PCR amplification (when too numerous or when occurring at the 3′ end of the primer), three different L1 downstream primers (PA1 to PA6) were designed and alternatively used. Specific L1 downstream and upstream primers were designed for the L1PA7 and L1PA8 subfamilies. Primer sequences, mismatch number between L1 primer/L1 sequence, and PCR product amplification sizes and results are given in Supplementary Material, Table S3. As previously described, 1 μg of total RNA was treated with DNase I and reverse transcribed with SuperScript III using random hexamers. For each sample, a control was prepared without reverse transcriptase. PCR amplifications were performed as previously described, using the relevant LCT-US primer and L1 downstream or upstream primer. In the PCR program, the extension time at 72°C was adjusted in function of the expected amplification product size (40 s and 1 min for L1 downstream and L1 upstream primers, respectively). As all tested chimeras corresponded to continuous transcription events from the L1 sequence and the adjacent unique sequence, genomic DNA was used as positive control and as size marker. PCR products were analyzed on 1.5% agarose gel.

### Splicing analysis

Three parameters were taken into account to identify splicing events in chimeras: (1) the distance between the start position of the unique sequence and the end position of the L1 sequence of each chimera after alignment (R1–R2 distance). A negative or null distance indicates an overlap or a juxtaposition of both sequences, and implies that these chimeras have been identified in multiple reads (either in one or different samples). A distance between 1 and 200 bp is concordant with the size selection made during the library construction (i.e. 280–340 bp) and often relevant of chimeras identified in few reads in all samples. A distance >500 bp may suggest at least one splicing event between the L1 and unique sequence; (2) the LCT size. Chimeras with a size >1000 bp, even if their R1–R2 distance is negative, can present evidences of splicing event(s), because the unique sequence is very large (>700 bp) due to concatenation of multiple reads by CLIFinder; (3) the occurrence of splice junctions identified by TopHat in the RNA-seq data in the ±200 bp region around the L1 start position. One of these parameters was considered sufficient to conclude that a LCT was spliced. LCT with R1-R2 distance between 200 and 499 bp and a size between 600 and 999 bp were considered to have an undetermined spliced status.

### RT-PCR analysis to determine the polyA and non-polyA status of LCTs

Total RNA from the glioma samples GS#1 and GS#2 was treated with DNase I and reverse transcribed with SuperScript III using 2.5 μm oligod(T)n primer (Invitrogen) to specifically target polyA mRNA transcripts. Then, 2 μl of diluted (1:10) cDNA was used for PCR amplification using the LCT-US and L1 common primers.

Alternatively, 3 μg of total RNA was treated with DNase I and then with 2 U of PolyU polymerase in the presence of 0.5 mm UTP, 1X Buffer and 40 U RNase inhibitor. The resulting poly-uridinylated RNA was purified on RNeasy columns (Qiagen), concentrated after ethanol precipitation, and 1 μg of PolyU-RNA was reverse transcribed using 0.6 μm of oligod(A)n primer (Eurogentec) and Superscript III (Invitrogen). Then, 2 μl of diluted (1:10) cDNAs was PCR amplified with the LCT-US and L1 common primers.

### Expression analysis of LCTs and associated genes in gliomas by microfluidic RT-qPCR

500 ng of total RNA was treated with DNase I (Promega) and divided in two independent aliquots (250 ng RNA/each) for reverse transcription using a random hexamer primer (Invitrogen). Then, first strand cDNA was pre-amplified (14 cycles) with the pool of primers used for the microfluidic PCR assays. Primer sequences and qPCR Efficiency (E) are given in Supplementary Material, Table S3. All primers used to quantify host gene expression are positioned upstream of the LCT TSS in L1. qPCR assays were performed and validated using Fluidigm 96.96 Dynamic Arrays and the Biomark HD system (Fluidigm) according to the manufacturer’s instructions. LCTs and host genes were quantified in separate arrays. The relative expression level was quantified as *R* = (*E*_TOI_^–CtTOI^)/(geometric mean *E*_HK_—^CtHK^) with *E* = PCR efficiency of each primer pair, TOI = Transcript Of Interest (i.e*.* chimera or gene to be quantified), and HK = housekeeping genes (i.e*. TBP*, *RPL13A* and *PPIA*) used to normalize transcript expression. For each sample, experiments were done in duplicate using the two independent RT reactions.

Differences in the expression levels of the LCT or associated gene between tumor and control samples were assessed with the one-way ANOVA and post-hoc Mann–Whitney test, if applicable. Significant *P*-values were adjusted with the Bonferroni correction. Correlation analyses between intragenic LCT and the associated gene expression levels were performed using Spearman’s correlation.

### Chromatin analysis at recent L1 loci

L1 positions and classifications were retrieved from the CLIFinder output. Based on these coordinates, chromatin analyses were done with the computeMatrix scale-regions tools from the deeptools suite (Version 3.1.3) (Parameters: —beforeRegionStartLength 1000, —regionBodyLength 1000, —afterRegionStartLength 1000, —skipZeros) and chromatin data coverages extracted from GeoDataset in WIG format (for Brain input: GSM669971 and GSM773019; for GSC input: GSM1121871 and GSM1121861; for Brain H3K36me3: GSM670002 and GSM916041; for GSC H3K36me3: GSM1121868 and GSM1121858; for Brain H3K9me3: GSM916034 and GSM773017; for Brain H3K27ac: GSM773020 and GSM916035; for Brain H3K4me1: GSM669962 and GSM916039; for Brain H3K4me3: GSM670022 and GSM916040; for Brain H3K27me3: GSM916038 and GSM669913). These WIG files were converted into binary format with the wigToBigWig program from the UCSC server, to be compatible with the computeMatrix program prerequisites. Chromatin profiles surrounding the L1 start and end positions (±1 kb) that corresponded to the mean chromatin coverage over the set of genomic regions were generated using the compute Matrix output and R program.

### Quantitative analysis of DNA methylation by qPCR

The qAMP approach relies on the use of methylation-sensitive (e.g. *Hha*I or *Hpa*II) and methylation-dependent (e.g*. McrBC*) restriction enzymes. DNA was extracted from frozen samples (21 *IDHwt* glioma and 7 control brain samples) using the QIAamp DNA Mini Kit (Qiagen) according to the manufacturer’s recommendations. For each DNA sample, four 200 ng DNA aliquots were prepared. Three were incubated with 5 U of *Hha*I, *Hpa*II, or *McrBC* (NEB) at 37°C for 2 h. The fourth aliquot was incubated only with buffer (negative control). The published qAMP protocol was slightly modified by adding a restriction enzyme inactivation step by incubation with proteinase K (0.4 mg/μl) at 40°C for 30 min, followed by its inactivation at 95°C for 10 min. The obtained PCR templates were 8-fold diluted in water and stored at −20°C. PCR primers covered approximately the first 300 bp of each L1 promoter in which the restriction sites for each of the three enzymes used are present. L1 primers were designed using the Primer 3 software and combined to the already described LCT US primer. Additionally, primers for control regions with different DNA methylation levels were designed: (1) a control region that does not contain restriction sites for the three enzymes; (2) the promoter of the imprinted gene *PEG10* that is hemi-methylated and (3) the *GAPDH* promoter region that in our samples, has a methylation level <10%. All primers used for the qAMP experiments are given in Supplementary Material, Table S3. Finally, artificially unmethylated (0% methylation) and fully methylated (100%) genomes were prepared by whole genome PCR amplification with the Repli-G Mini Kit (Qiagen) and artificial methylation with the *Sss*I enzyme (NEB). These two DNA templates were mixed to obtain a standard curve with theoretical DNA methylation levels of 0, 30, 70 and 100%. The efficiency of each primer pair was evaluated using 3 log_10_ gDNA concentrations. Only primer pairs with an efficiency ≥1.85 and a standard curve corresponding to the expected theoretical DNA methylation percentages were retained. The DNA methylation index of each sample was then evaluated by qAMP on a LightCyclerR©480II (Roche). Four μl of each DNA (sham, *Hpa*II-, *Hha*I- and *McrBC*-digested) was mixed with 1× SYBR Green Master Mix (Roche) and 0.5 μm forward and reverse primers in a final volume of 10 μl. The PCR program was: 95°C for 5 min, and then 40 cycles (95°C for 15 s, 60°C for 15 s, 72°C for 15 s), and 72°C for 5 min. The DNA methylation index of each sample was calculated using the amplification data obtained for the three DNA samples digested with the restriction enzymes and as previously described using the ∆Ct values (Ct enzyme—Ct sham) (29). Differences in methylation index levels between glioma and control samples were assessed with the one-way ANOVA and post-hoc Mann–Whitney test, when applicable. Significant *P*-values were adjusted using the Bonferroni correction. Correlation analyses between L1 methylation index and LCT expression level were done using the Spearman’s correlation.

## Supplementary Material

Pinson_et_al_Supp_Fig_ddac056

Pinson_et_al_Table_S1_ddac056

Pinson_et_al_Table_S2_ddac056

Pinson_et_al_Table_S3_ddac056
