# Supplementary material for: L1 chimeric transcripts are expressed in healthy brain and their deregulation in glioma follows that of their host locus
Source: Hum Mol Genet. 2022 Mar 17;31(15):2606–22. doi: 10.1093/hmg/ddac056 (PMC9396940; doi:10.1093/hmg/ddac056)
Supplement: Pinson_et_al_Supp_Fig_ddac056 [file pinson_et_al_supp_fig_ddac056.pptx]

## Slide 1
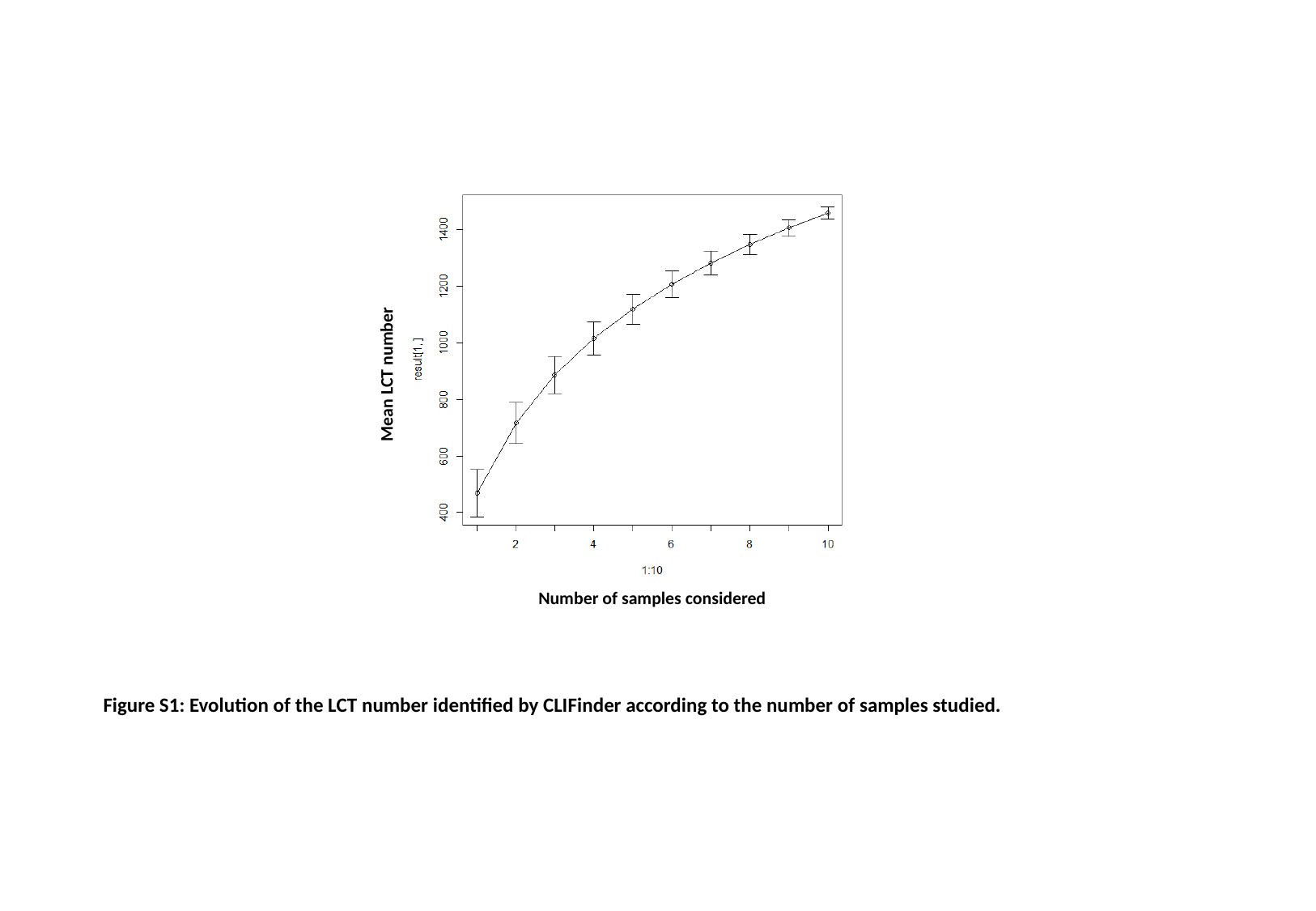

Mean LCT number
Number of samples considered
Figure S1: Evolution of the LCT number identified by CLIFinder according to the number of samples studied.

## Slide 2
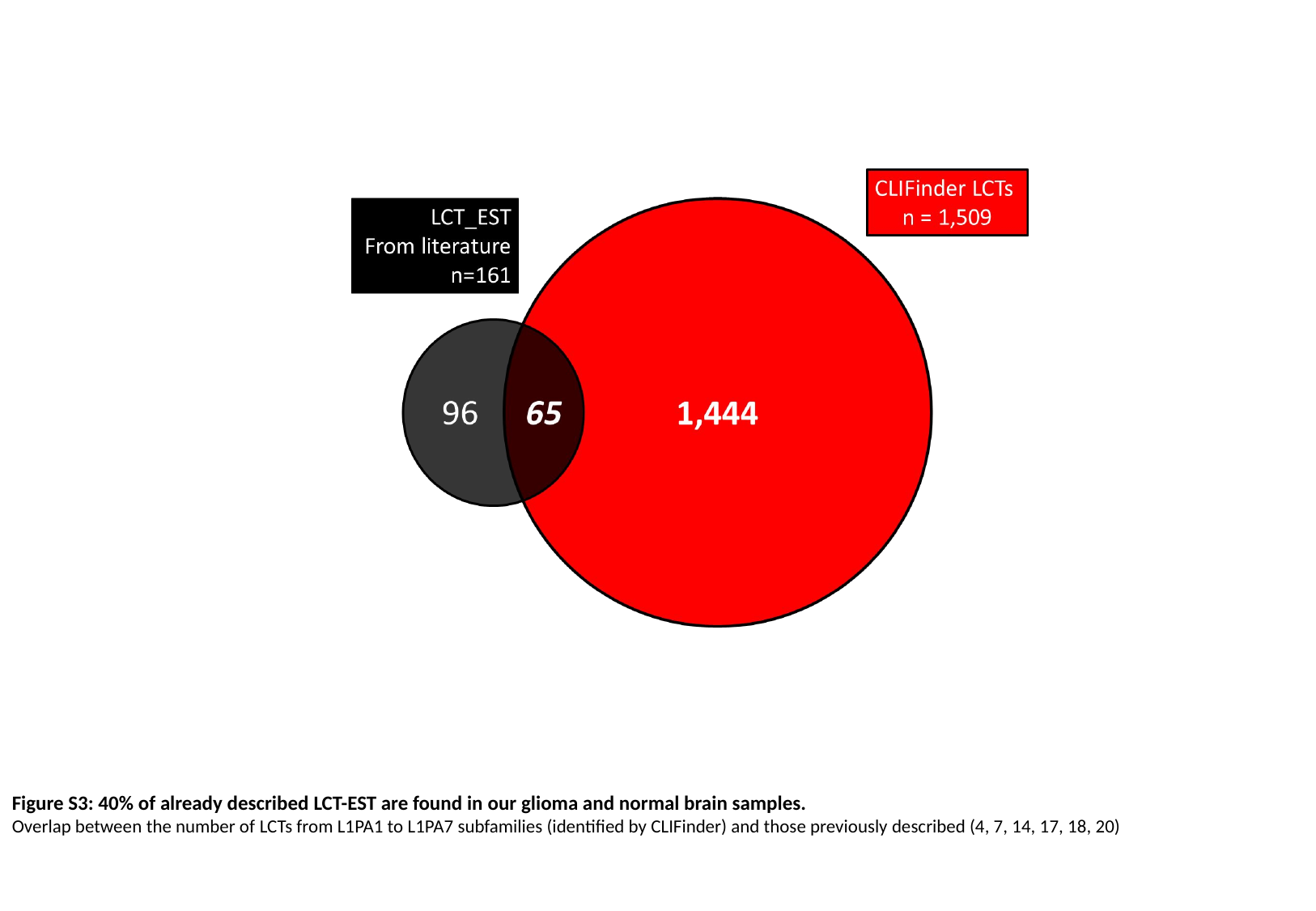

Figure S3: 40% of already described LCT-EST are found in our glioma and normal brain samples.
Overlap between the number of LCTs from L1PA1 to L1PA7 subfamilies (identified by CLIFinder) and those previously described (4, 7, 14, 17, 18, 20)

## Slide 3
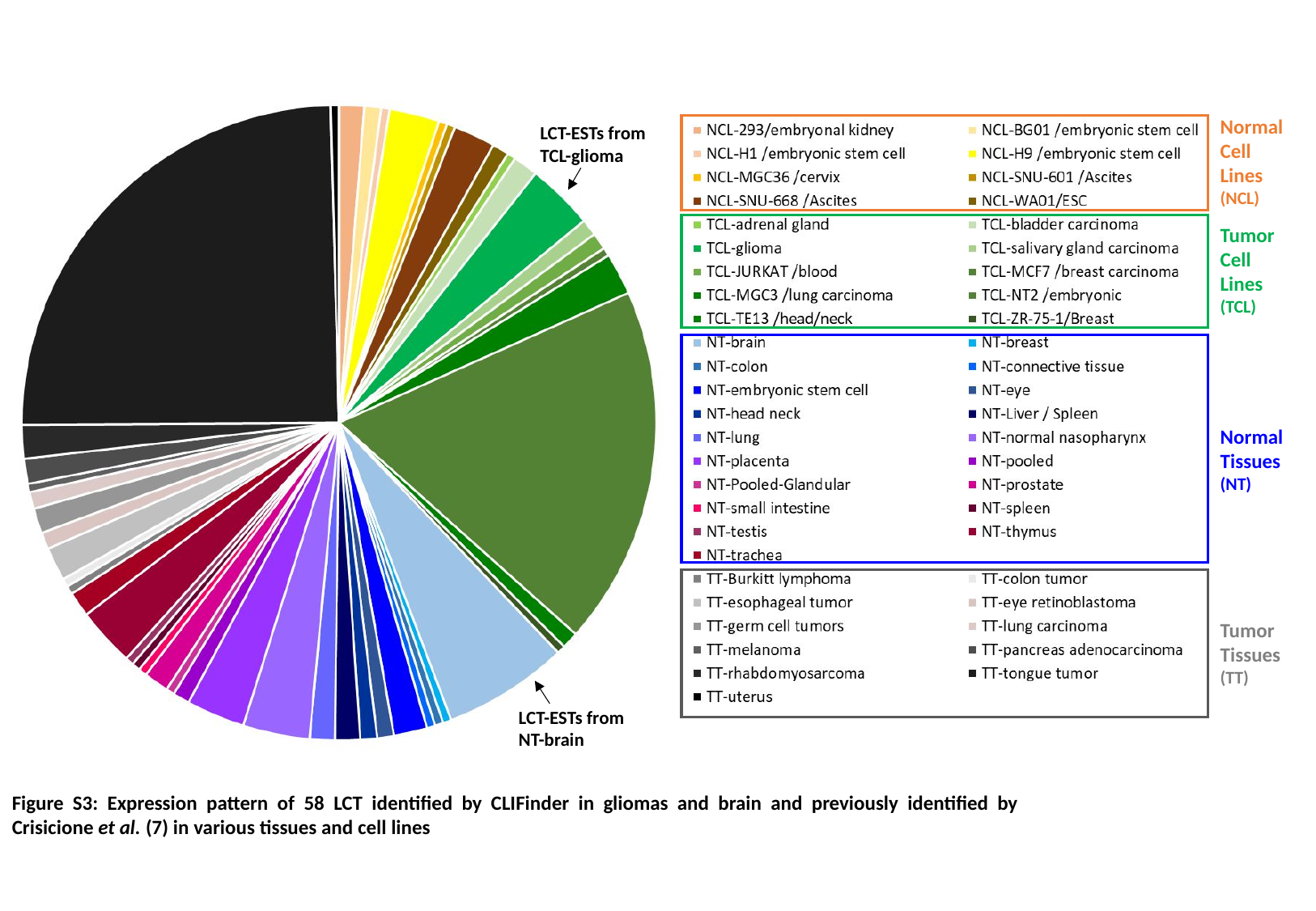

Normal Cell Lines
(NCL)
LCT-ESTs from
TCL-glioma
Tumor Cell Lines
(TCL)
Normal Tissues
(NT)
Tumor Tissues
(TT)
LCT-ESTs from
NT-brain
Figure S3: Expression pattern of 58 LCT identified by CLIFinder in gliomas and brain and previously identified by Crisicione et al. (7) in various tissues and cell lines

## Slide 4
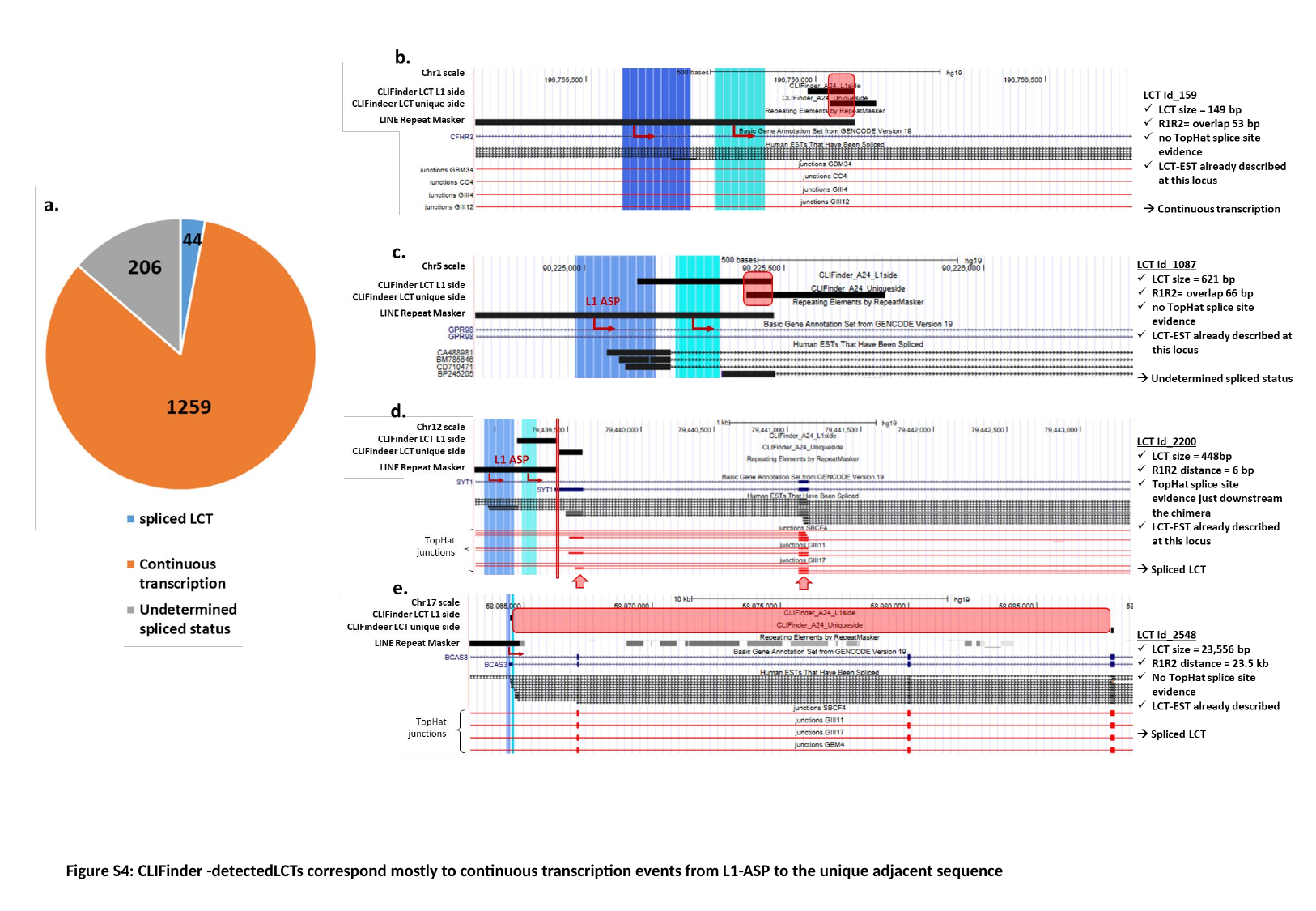

Figure S4: CLIFinder -detectedLCTs correspond mostly to continuous transcription events from L1-ASP to the unique adjacent sequence

## Slide 5
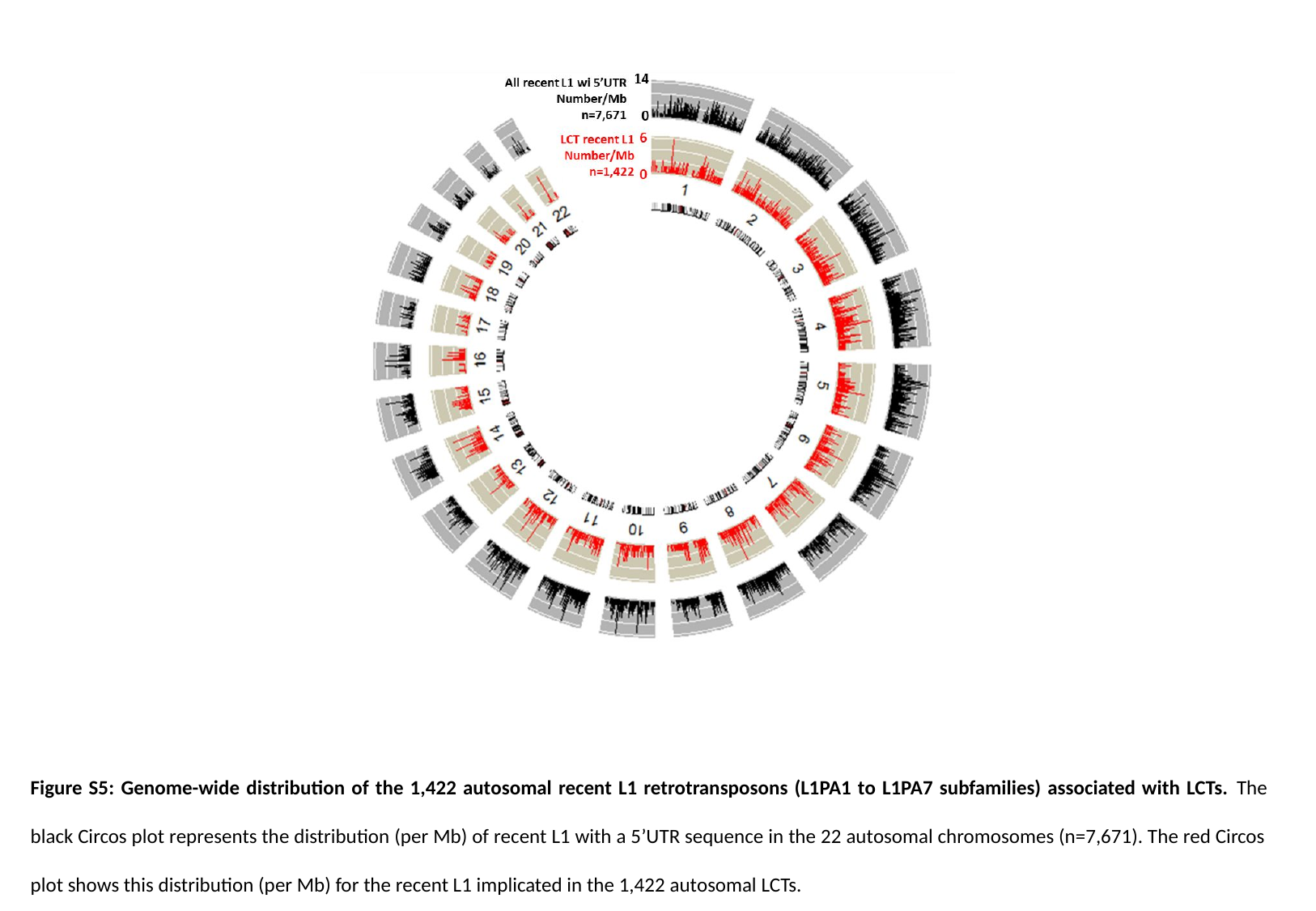

Figure S5: Genome-wide distribution of the 1,422 autosomal recent L1 retrotransposons (L1PA1 to L1PA7 subfamilies) associated with LCTs. The black Circos plot represents the distribution (per Mb) of recent L1 with a 5’UTR sequence in the 22 autosomal chromosomes (n=7,671). The red Circos plot shows this distribution (per Mb) for the recent L1 implicated in the 1,422 autosomal LCTs.

## Slide 6
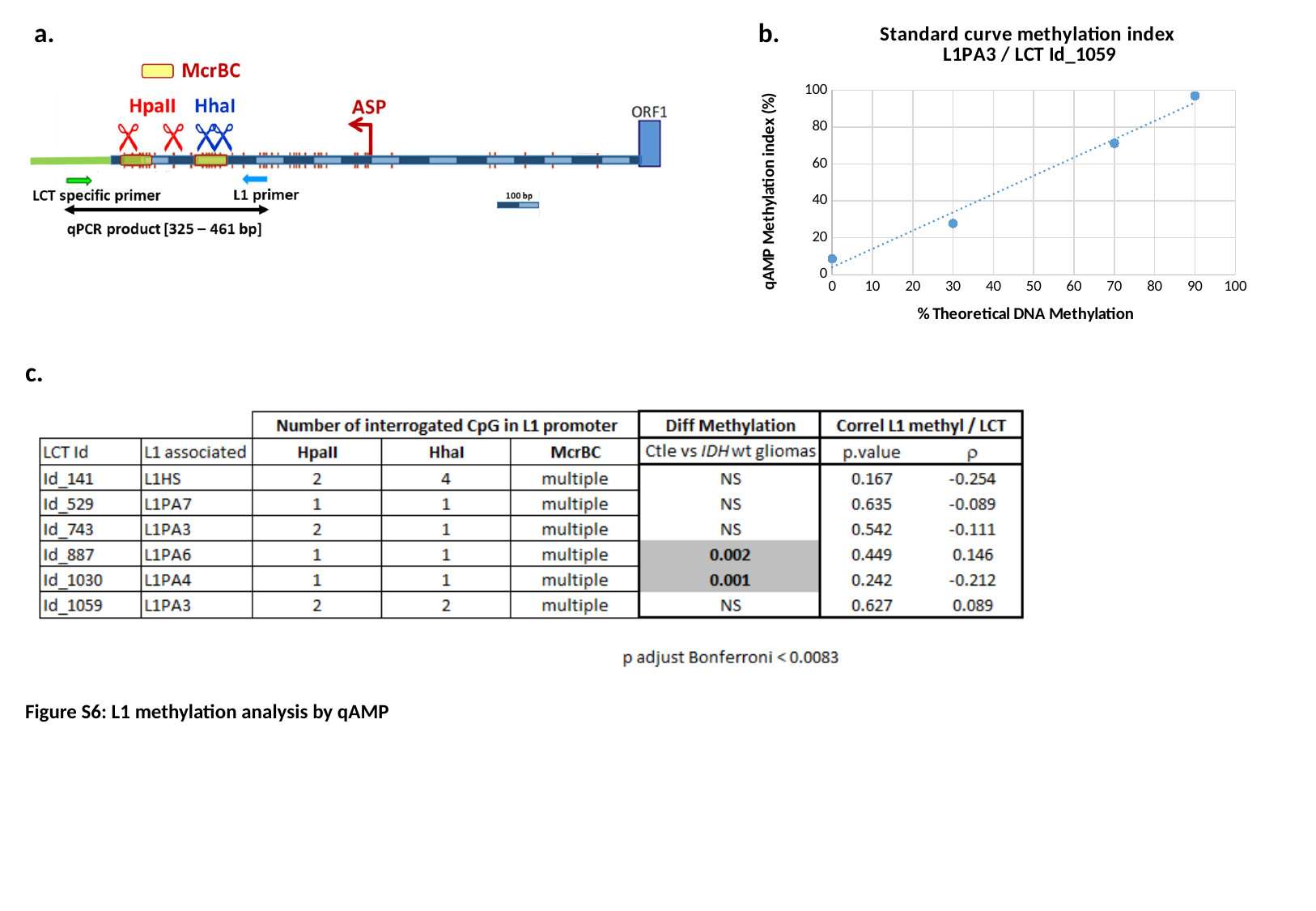

a.
### Chart: Standard curve methylation index
L1PA3 / LCT Id_1059
| Category | |
|---|---|b.
c.
Figure S6: L1 methylation analysis by qAMP

## Slide 7
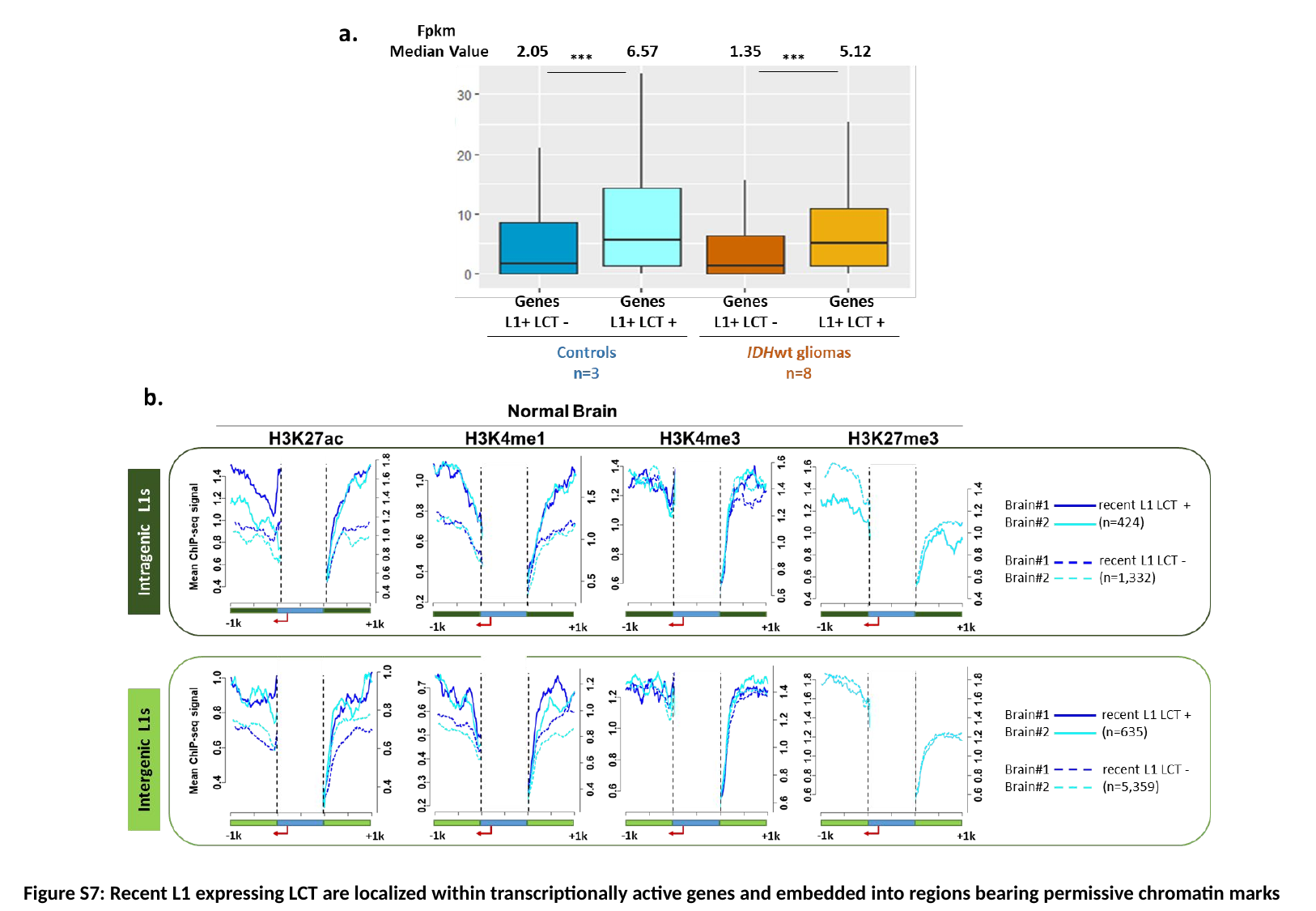

Figure S7: Recent L1 expressing LCT are localized within transcriptionally active genes and embedded into regions bearing permissive chromatin marks
